# Easier surveillance of climate-related health vulnerabilities through a Web-based spatial OLAP application

**DOI:** 10.1186/1476-072X-8-18

**Published:** 2009-04-03

**Authors:** Eveline Bernier, Pierre Gosselin, Thierry Badard, Yvan Bédard

**Affiliations:** 1 Center for Research in Geomatics (CRG), Université Laval, Québec, Canada; 2 NSERC Industrial Research Chair on Geospatial Databases for Decision Support, Université Laval, Québec, Canada; 3 Institut National de Santé Publique du Québec (INSPQ), Québec, Canada; 4 Ouranos, Montréal, Canada

## Abstract

**Background:**

Climate change has a significant impact on population health. Population vulnerabilities depend on several determinants of different types, including biological, psychological, environmental, social and economic ones. Surveillance of climate-related health vulnerabilities must take into account these different factors, their interdependence, as well as their inherent spatial and temporal aspects on several scales, for informed analyses. Currently used technology includes commercial off-the-shelf Geographic Information Systems (GIS) and Database Management Systems with spatial extensions. It has been widely recognized that such OLTP (On-Line Transaction Processing) systems were not designed to support complex, multi-temporal and multi-scale analysis as required above. On-Line Analytical Processing (OLAP) is central to the field known as BI (Business Intelligence), a key field for such decision-support systems. In the last few years, we have seen a few projects that combine OLAP and GIS to improve spatio-temporal analysis and geographic knowledge discovery. This has given rise to SOLAP (Spatial OLAP) and a new research area. This paper presents how SOLAP and climate-related health vulnerability data were investigated and combined to facilitate surveillance.

**Results:**

Based on recent spatial decision-support technologies, this paper presents a spatio-temporal web-based application that goes beyond GIS applications with regard to speed, ease of use, and interactive analysis capabilities. It supports the multi-scale exploration and analysis of integrated socio-economic, health and environmental geospatial data over several periods. This project was meant to validate the potential of recent technologies to contribute to a better understanding of the interactions between public health and climate change, and to facilitate future decision-making by public health agencies and municipalities in Canada and elsewhere. The project also aimed at integrating an initial collection of geo-referenced multi-scale indicators that were identified by Canadian specialists and end-users as relevant for the surveillance of the public health impacts of climate change. This system was developed in a multidisciplinary context involving researchers, policy makers and practitioners, using BI and web-mapping concepts (more particularly SOLAP technologies), while exploring new solutions for frequent automatic updating of data and for providing contextual warnings for users (to minimize the risk of data misinterpretation). According to the project participants, the final system succeeds in facilitating surveillance activities in a way not achievable with today's GIS. Regarding the experiments on frequent automatic updating and contextual user warnings, the results obtained indicate that these are meaningful and achievable goals but they still require research and development for their successful implementation in the context of surveillance and multiple organizations.

**Conclusion:**

Surveillance of climate-related health vulnerabilities may be more efficiently supported using a combination of BI and GIS concepts, and more specifically, SOLAP technologies (in that it facilitates and accelerates multi-scale spatial and temporal analysis to a point where a user can maintain an uninterrupted train of thought by focussing on "what" she/he wants (not on "how" to get it) and always obtain instant answers, including to the most complex queries that take minutes or hours with OLTP systems (e.g., aggregated, temporal, comparative)). The developed system respects Newell's cognitive band of 10 seconds when performing knowledge discovery (exploring data, looking for hypotheses, validating models). The developed system provides new operators for easily and rapidly exploring multidimensional data at different levels of granularity, for different regions and epochs, and for visualizing the results in synchronized maps, tables and charts. It is naturally adapted to deal with multiscale indicators such as those used in the surveillance community, as confirmed by this project's end-users.

## Background

Global warming is now a widely recognized fact, and scientists throughout the world are working to understand its impacts on society [1]. The warming climate will bring more extreme weather events, more frequent and severe heat waves, new infectious diseases and ideal conditions for their spread, to name a few consequences [2]. Indeed, the present situation is both worrying and very complex: everybody is vulnerable to climate change, but the level of this vulnerability differs according to several different aspects: psychological, biological, social, cultural, economic, environmental and, of course, climatic. In 2004, the World Health Organization [3] recognized that national health agencies should play a major role in the management of and adaptation to the potential negative impacts of these events on population health and well-being, notably through improved surveillance systems.

This surveillance is usually done by indicators of the health impacts of thermal extremes and other extreme climate events (ECE); water- and food-borne contamination; vector-borne and zoonotic contamination; ultraviolet radiation. Other health impacts are related to the exacerbation of these impacts by climate on vulnerable sub-populations such as homeless, disabled or chronically ill persons. Other indirect impacts are related to the loss of income and productivity or the social disruption brought on by ECE [4]. To be efficient, surveillance of climate-related health vulnerabilities must take into account a wide variety of factors influencing health outcomes, as well as their associated spatial and temporal aspects [2].

Such analyses can rapidly become complex and must rely on appropriate tools and technologies that will support data analysis and exploration in an effective way. Geographic information systems (GIS) have made their mark in the health field for many years now [5]. They are powerful tools for detailed spatial data analysis, spatial processes (e.g., buffering, route finding, distance measurements), and spatial data updating, etc. However, their transactional nature is not meant to support analytical needs, which mostly require summarized information, aggregated data, trends analysis, spatio-temporal comparisons, interactive exploration of data, geographic knowledge discovery in large amounts of data, etc. Consequently, supporting such analyses with GIS or DBMS requires major efforts. On the other hand, these needs are typically addressed by technologies that fall within the field called "*Business Intelligence*" (BI), which mostly involves OLAP, data mining and dashboard technologies. BI technologies are built to support complex summarized multi-scale cross-tabbed queries for knowledge discovery rather than data transactions [6]. Although they perform better for complex analysis, they rely on transactional systems to feed them since the latter were developed mainly to store, structure, validate, and disseminate data (when compared to BI types of technologies) [7-10]. BI systems were not designed to replace transactional systems; they were built as add-ons specialized for solving the inherent decision-support weaknesses of transactional systems with regard to temporal data (e.g., for trends analysis), cross-tabbed data (e.g., for correlation analysis), and multi-scale summarized data (e.g., for comparison analysis). BI technology relies on a different data structure called the *hypercube *(or *datacube*) [6,11-13], and consequently requires extraction of a copy of the source data from the transactional systems, transformation of source data (cleaning, integration, summarizing), and loading of the result into a read-only system (e.g., data warehouse, datamart, OLAP server). This is called the ETL (Extract, Transform, Load) process [14]. The hypercube structure has its roots in the field of statistics, where it is a common task to analyze data in 2-D or 3-D tables as well as in n-D tables (multidimensional tables or hypercubes) [12]. The weakness of traditional transaction-based systems (e.g., DBMS – Data Base Management Systems, GIS) for such multidimensional tables, especially when they involve n hierarchical dimensions (e.g., property-city-state-country; day-month-quarter-year) was recognized by the database community in the early 1990s and led to the development of OLAP (On-Line Analytical Processing, a term coined by Codd [15] to clearly indicate the difference from On-Line Transaction Processing (OLTP). Over the years, we have also witnessed the arrival of related technologies such as dashboard, ETL-specific tools, data mining, datamart and data warehousing under the umbrella of BI technologies. While BI technologies have become common practice in several organizations, it is only in the last few years that commercial software that bridges BI and geospatial technologies, such as Spatial OLAP, has appeared [5,16]. These technologies are not meant to replace GIS and web-mapping applications, but rather to facilitate new analyses of existing data stored in current systems. It is beyond the goal of the present paper to define the underlying concepts involved in OLAP/SOLAP since a rich literature already exists and several companies offer products and services (cf., data warehousing, multidimensional databases, analytical processing, and business intelligence).

The system presented in this paper is based on the Spatial OLAP (SOLAP) technology. SOLAP has emerged from the combination of OLAP functionalities with GIS functionalities. For over a decade, OLAP tools have proven their worth in the BI field, but have lacked spatial capabilities. The recent availability of commercial SOLAP technology has led to the investigation of its potential for the analysis of climate change impacts on population health with the help of multi-scale surveillance indicators developed by a group of specialists and users. SOLAP technology is based on the OLAP philosophy and provides ways that are more intuitive and rapid for exploring interactively and analyzing large volumes of cross-tabbed multi-scale spatio-temporal data than what is offered today by GIS packages and DBMS with spatial extensions. Using simple operators, it allows users to rapidly navigate through the data using tables, charts or cartographic displays that can be synchronized or not. The user can explore within seconds several analysis themes and immediately see interactions (e.g., age, sex, income, region, year), at different levels of detail (LoD) such as local, regional, national and global. As opposed to GIS tools, SOLAP is specifically intended to support the analysis needs of decision-makers. Its underlying multidimensional structure (i.e., spatial datacube) is well adapted to complex queries, temporal analyses, aggregated views, etc. Despite the complexity of the query and display (e.g., 1 summarized table covering 5 years with one histogram + one map per year), it typically provides response times within 1 second (and always well below Newell's cognitive band of 10 seconds [17]) and does not interrupt the user's train of thought. There is no need to master any query languages or to know the underlying database's structure. Given that navigation is entirely done via mouse clicks, SOLAP tools are often regarded as *keyboard-less GIS *although they offer improved interactive exploration capabilities. While one could assume that similar results can be obtained with GIS technology, it would require several months of development as there are no built-in operators for interactive exploration of multi-scale cross-tabbed data, and their inherent transaction-oriented data structure would require several table joins (a computer intensive process) and never achieve the necessary performance. On the other hand, SOLAP data structures and operators are built especially for such work and allow an increase in performance of at least two to three orders of magnitude (10^2 ^to 10^3 ^times faster) while offering a more intuitive user interface (one only has to click with the mouse on the desired data) and greater flexibility (while GIS requires the use of either SQL sentences, query forms or limited lists of pre-programmed queries).

This project was meant to validate the potential of those recent SOLAP technologies to help better understand the interactions between public health and climate change, and to facilitate future decision making by public health agencies and municipalities in Canada and elsewhere. The application that is presented here was developed in the context of an 18-month project funded by the Canadian Network of Centres of Excellence in Geomatics – GEOIDE – in 2007–2008 and brought together researchers, policy makers and practitioners from different spheres, including health, geomatics, statistics and climate change. As explained in the next section, they participated in the development of geo-referenced surveillance indicators, provided data, and analyzed the developed system.

## Results

### SOLAP, a new technology for exploring spatio-temporal surveillance data

**Figure 1:**
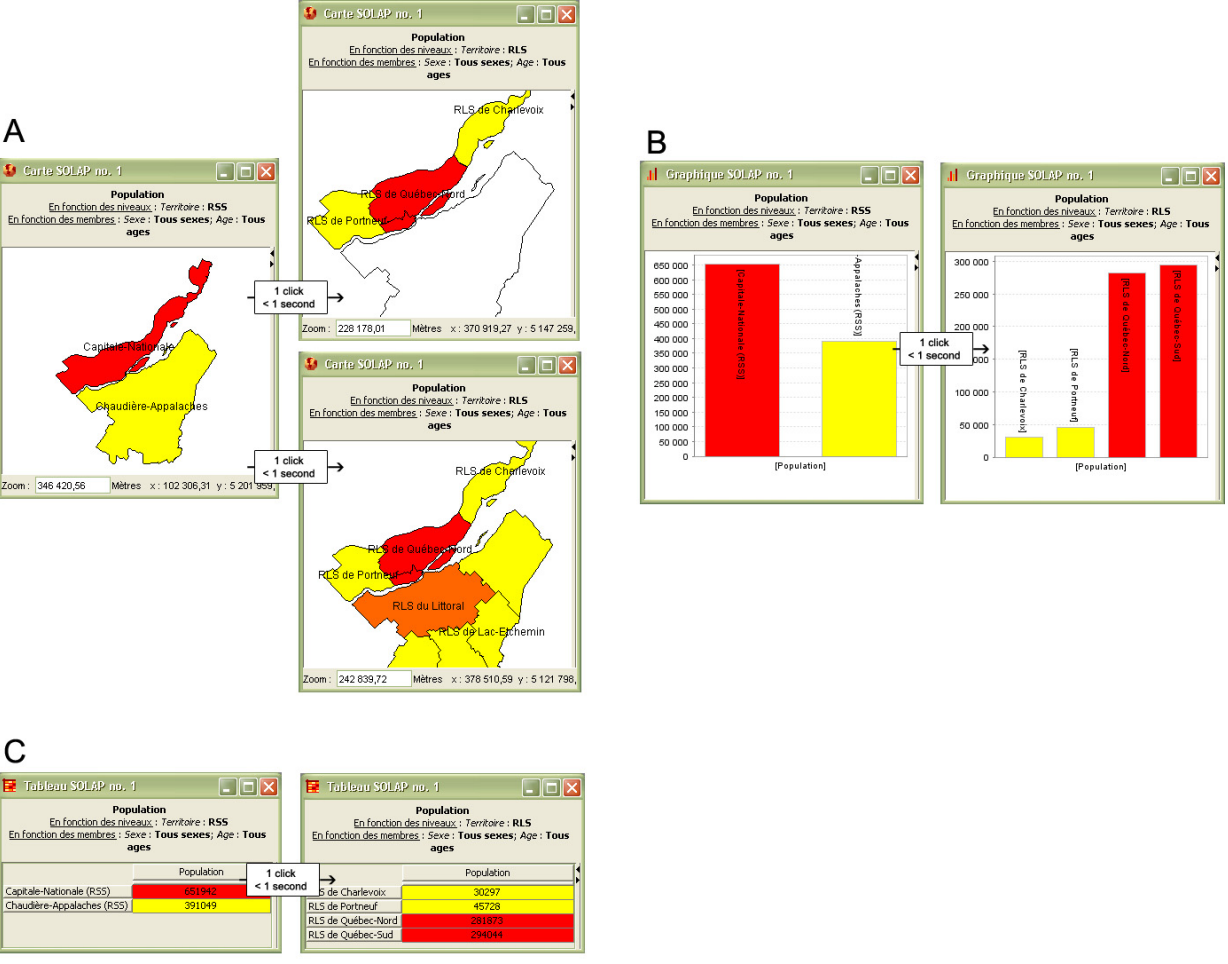
**Exploring the data through the drill functionalities**. The drill functionalities can be used in any of the three types of displays (cartographic, chart and tabular). Navigating through different LoDs is simply done by clicking directly on an element or a group of elements. The automatic legends have been removed for better clarity. **Section A**: (top) Spatial drill-down on a specific member. The result shows only the number of people for the sub-regions included in the selected member. (bottom) Spatial drill-down on a specific LoD. The result shows the number of people for all the members of the next LoD, i.e., all the sub-regions. **Section B**: Drill-down on a specific member, i.e., a bar of the bar chart. The result shows the number of people for the sub-regions included in the selected member. **Section C**: Drill-down on a specific member, i.e., a row in the table. The result shows the number of people for the sub-regions included in the selected member.

The examples presented later in this section are only a few possibilities of analyses that can be carried out more efficiently than with existing GIS. The user's facility in interacting with the data allows a multitude of analyses to be rapidly performed, phenomena to be compared in space and especially in time, information to be drilled down or rolled up to obtain a detailed or a general portrait of a situation, simple and cross-analyses to be performed, etc. In fact, all the data navigation is instantaneous and done using the mouse. For example, the user selects the themes to be analyzed (e.g., sex, age and region), the level of detail to which each theme should be analyzed to begin (e.g., for all sexes or only for women, for all ages or for certain classes, for each health region or for all of Québec province) and the display format (tables and/or charts and/or maps, synchronized or not). The selection is simply done by dragging measures and analysis themes (called dimensions) to be analyzed into columns and rows (bottom left part of the interface). Navigation through the different LoDs is simply done using the *drill *operators (Figure 1). These operators give a more detailed LoD (drill-down) or a more global LoD (roll-up) of the information (descriptive and/or spatial and/or temporal). Such multi-level analyses are essential to adequately support decision-making, given that some trends or correlations may only appear at a certain LoD, or some risks only involve certain subpopulations. As in typical OLAP technology, the drill operators behave in the same way, regardless of the display type (e.g., simple click on a bar of a bar chart, on a sector of a pie chart, on a cell of a table, or on a spatial member of a map). For example, the spatial drill-down operator allows more detail to be obtained by clicking directly on a province on the map (i.e., on the spatial members) and information to be obtained about the socio-sanitary regions. This operation can be applied to all provinces at once (spatial drill-down on level), to a specific province (standard drill-down), or to a selected group of provinces (spatial drill-downs on members). The opposite operation (i.e., roll-up) allows a more general portrait to be obtained of the phenomenon to be studied, with a simple mouse click. Such operators are not provided by commercial GIS systems, except for the level drill, which has been developed in rare GIS applications.

**Figure 2:**
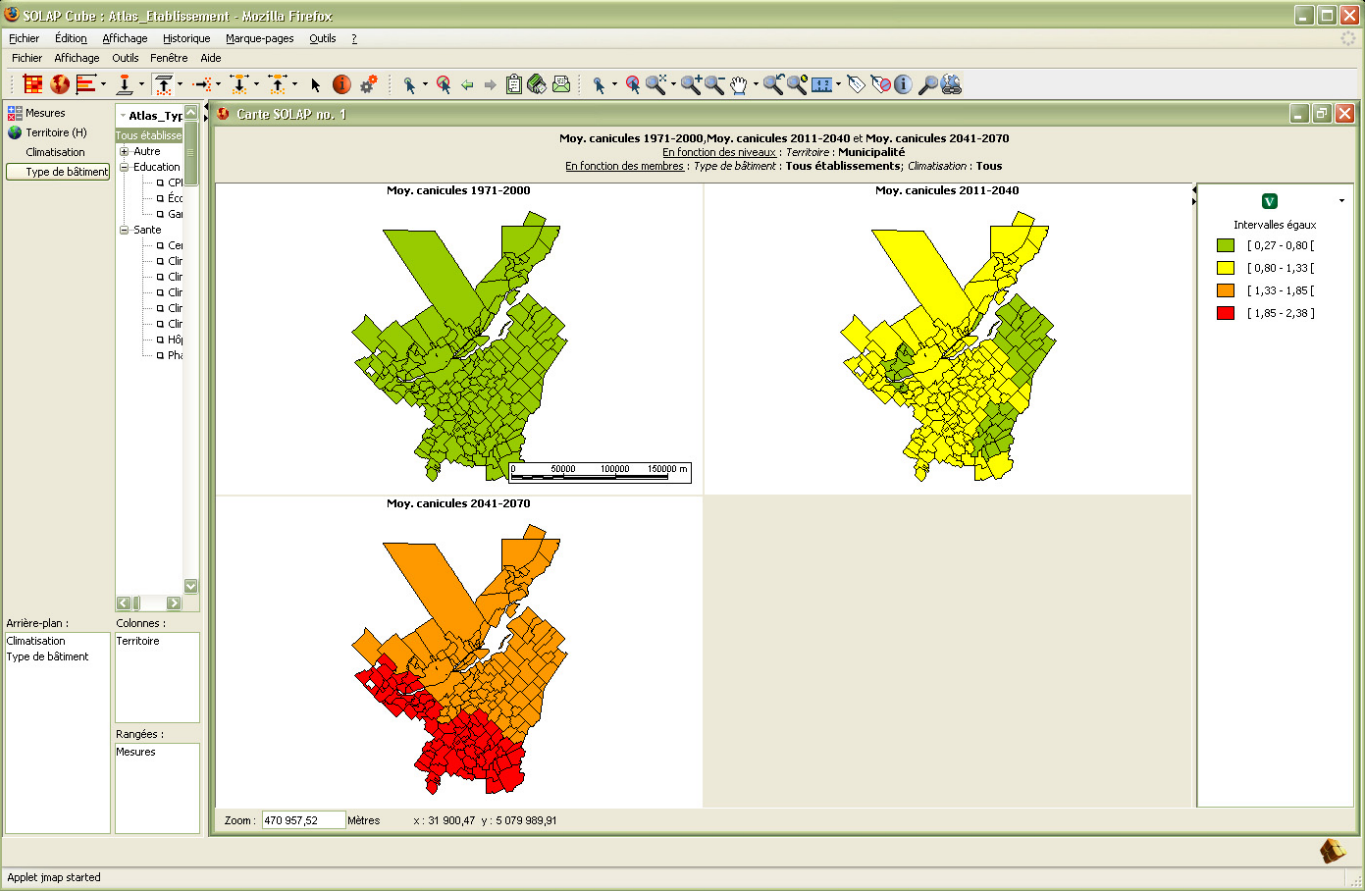
**Spatio-temporal evolution of heat waves using automatically built comparative multiple maps**. The first map (top left) is obtained from observed data from 1971–2000, the second map (top right) is obtained from prediction data for 2011–2040, and the third map (bottom) is obtained from prediction data for 2041–2070.

This instantaneous and easy navigation (mouse clicks) through the data allows the user to keep her/his train of thought during the analysis, even for typical Windows, Linux or MacOS-based laptop computers connected to the Internet via 802.11b wi-fi (such as the ones used in this project). Although such operations can be done using GIS, they would require several complex manipulations to obtain each level of detail, to know the underlying complex temporal database structure (table names, field names, etc.), to define SQL queries, to summarize the data to the desired LoD, to apply data classification and symbology, etc. These steps would have to be repeated for each LoD, each epoch, each query, and typically store the most useful ones in a list while leaving the remaining ones (the large majority of queries) for *ad hoc *query building. On the other hand, with the developed SOLAP system, spatio-temporal evolutions can be analyzed with a few mouse clicks and almost instantly. Figure 2 shows the evolution of heat waves using data from 2001 and prediction data for 2025 and 2050. Cartographic and semiology rules are also entirely managed by the application, so the user does not have to bother with selecting the visual variables (e.g., colours, patterns, line weight) during an analysis. Different displays for the same information are always one click away (e.g., tables, histograms, pie charts) and they can be synchronized or not according to the user's demand. When synchronized, one drill operation performed on one display automatically applies to the other displays, so each display keeps showing the same information. Every practitioner involved in the project witnessed the major increase in the quantity of queries that can be answered with the developed SOLAP application compared to their usual transactional system.

In short, the spatio-temporal Internet application that has been developed provides decision-makers with an innovative and powerful means for exploring, analyzing, comparing, cross-tabulating and seeing climate-related geo-referenced indicators in larger contexts. Compared to the typical commercial GIS, its user-friendly interface allows geographic knowledge discovery without the help of a software specialist. However, as previously mentioned, it is not intended to replace GIS in the management of spatial data; instead it adds new capabilities by reorganizing a copy of the GIS data and offering new operators and a user interface. The system developed in this project is based on the JMap^® ^Spatial OLAP solution that was developed at the Centre for Research in Geomatics at Laval University and commercialized by KHEOPS technology in Montréal [18]. This solution was the first commercial technology to integrate the geospatial dimension within a Business Intelligence environment. The current application is available through the Internet as a Java applet. Hence, users do not need specialized software to access the application. It only requires that the Java virtual machine be installed on the computer (which is free and can be downloaded from the Sun Microsystems web site). Furthermore, and contrary to the SOVAT system [19], which is specifically intended to support community health assessment data analysis [20], JMap^® ^Spatial OLAP is a generic solution that can be used in numerous application domains. The interface/functionalities remain the same, regardless of the data being explored; in addition, users can consult many datacubes for different groups of analysis through the same SOLAP user interface. Information about available commercial technologies, SOLAP concepts and SOLAP application examples is available online [21].

### Geo-referenced indicators for the surveillance of public health impacts of climate change

Population vulnerability to climate change depends on numerous factors. For example, elderly people with chronic respiratory and cardiac diseases who live alone in apartments located in mineralized urban areas (which are conducive to urban heat islands) are more exposed and vulnerable to heat waves than young and healthy people living in shaded rural areas. This situation needs to be known for today's situation as well as predicted for future situations in order to detect emerging trends. It must also be known for different areas and compared to more global situations to prioritize actions and better plan budgets. Surveillance tools should ideally take into account all these different factors along their needed levels of detail and epochs for "context-aware" analysis and decision-making. Vulnerability indicators are already being developed within the climate change community [22]. However, they are usually defined at a very global level of detail (i.e., usually country or large state) and are barely starting to include health preoccupations. Moreover, while national indicators can prove useful in influencing policies and budget allocation, they are generally not very useful at the local level where, for instance, practitioners need to organize emergency preparedness plans and know exactly where handicapped or sick seniors live if they want to either evacuate them if need be, or implement preventive measures to decrease risks. Inversely, relying solely on the latter detailed information does not provide the necessary global and comparative views for well-informed and context-aware decision-making, such as inter-regional budgetary allocation based on actual risks and needs.

This project led to a first set of geo-referenced indicators developed by field practitioners in mid-size cities. It was tested in the Québec City (Canada) metropolitan area (about 1,000,000 inhabitants) to monitor the local or regional impacts of global warming on public health (a subset of these indicators is presented in Appendix 1). These indicators were defined using a participatory approach with decision-makers (from the public health and civil protection fields, and from regional/municipal administrations) and include psychological, biological, social, cultural, economic, institutional, environmental and climatic aspects. Although they mainly focus on heat- and water-related risks and health impacts for the first version of the system, some of these geo-referenced indicators (notably those on pre-existing social vulnerabilities) are generic enough to be useful for other issues related to climate change, in the sense that vulnerabilities often share common factors such as age or socio-economic levels. Furthermore, despite being defined for medium- and long-term prevention contexts (next 10 years; up to 2050), they can become very useful in planning short-term emergency preparedness and risk prevention measures.

### A multitude of analyses one click away

With the application that has been developed, it is possible to obtain instantly the overall and detailed portraits of the population and building distribution over the territory. For instance, it allows areas to be identified where people are most vulnerable to climate change depending on several factors; population vulnerabilities to be compared according to sex or age; population distribution to be compared for different regions (from a global or local spatial perspective); the global increase in heat waves to be visualized over a 50-year horizon and this evolution to be compared for different regions at a more local level; the number of children in schools without air-conditioning to be evaluated and compared (at a regional level or at the school level) while taking into account urban heat spots; emergency measures to be better planned in case of flooding by identifying the buildings that are located in flooding areas while taking into account the average age of the population living in these areas; and so on, always with a few mouse clicks and within a few seconds. The paragraphs below describe three examples of analysis that can be carried out using the application.

**Figure 3:**
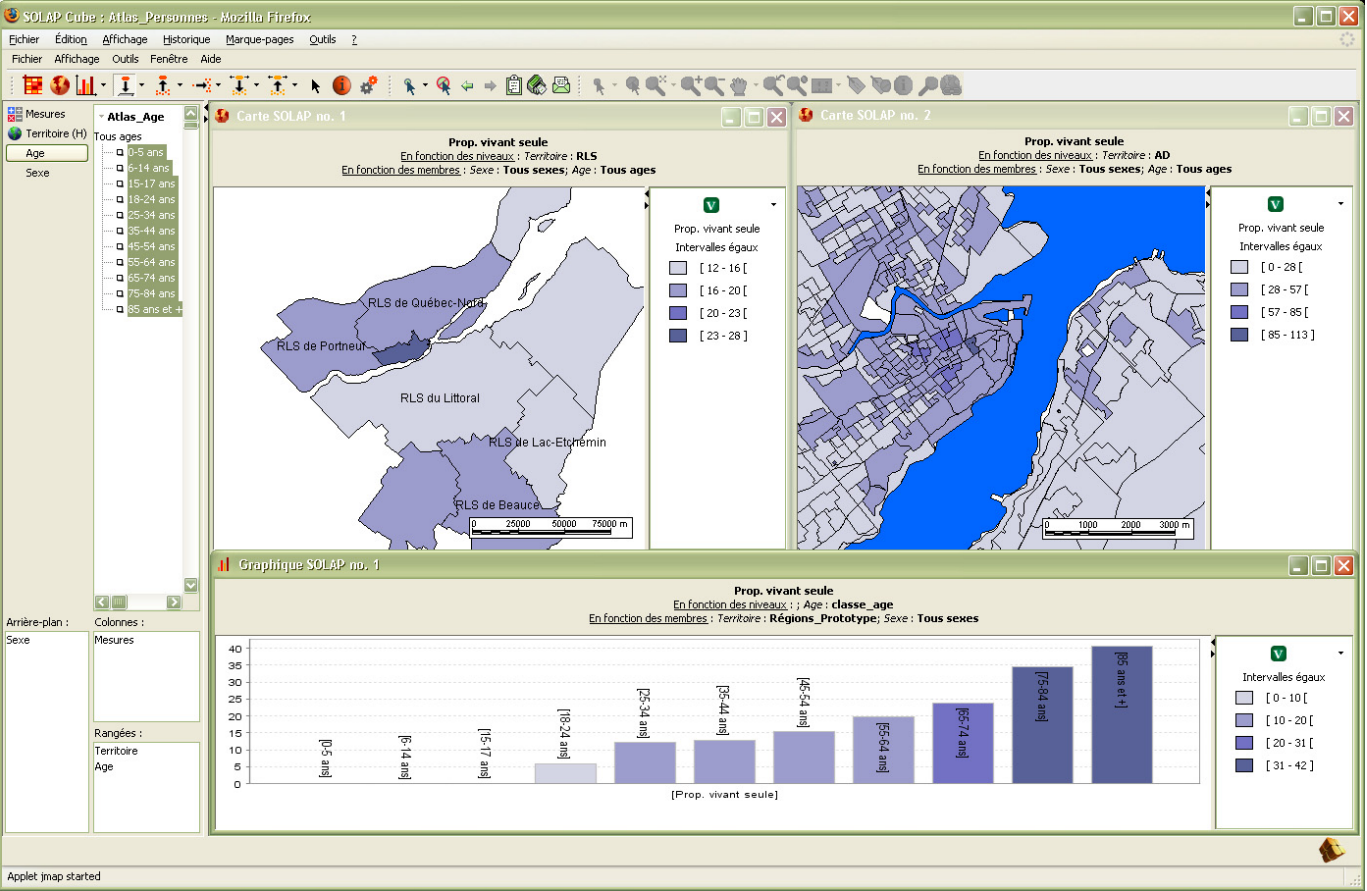
**Proportion of the population living alone**. Spatial representation of the proportion of the population living alone at two different LoDs (upper maps) and distribution of the population living alone by age group (table) for the entire territory.

**Figure 4:**
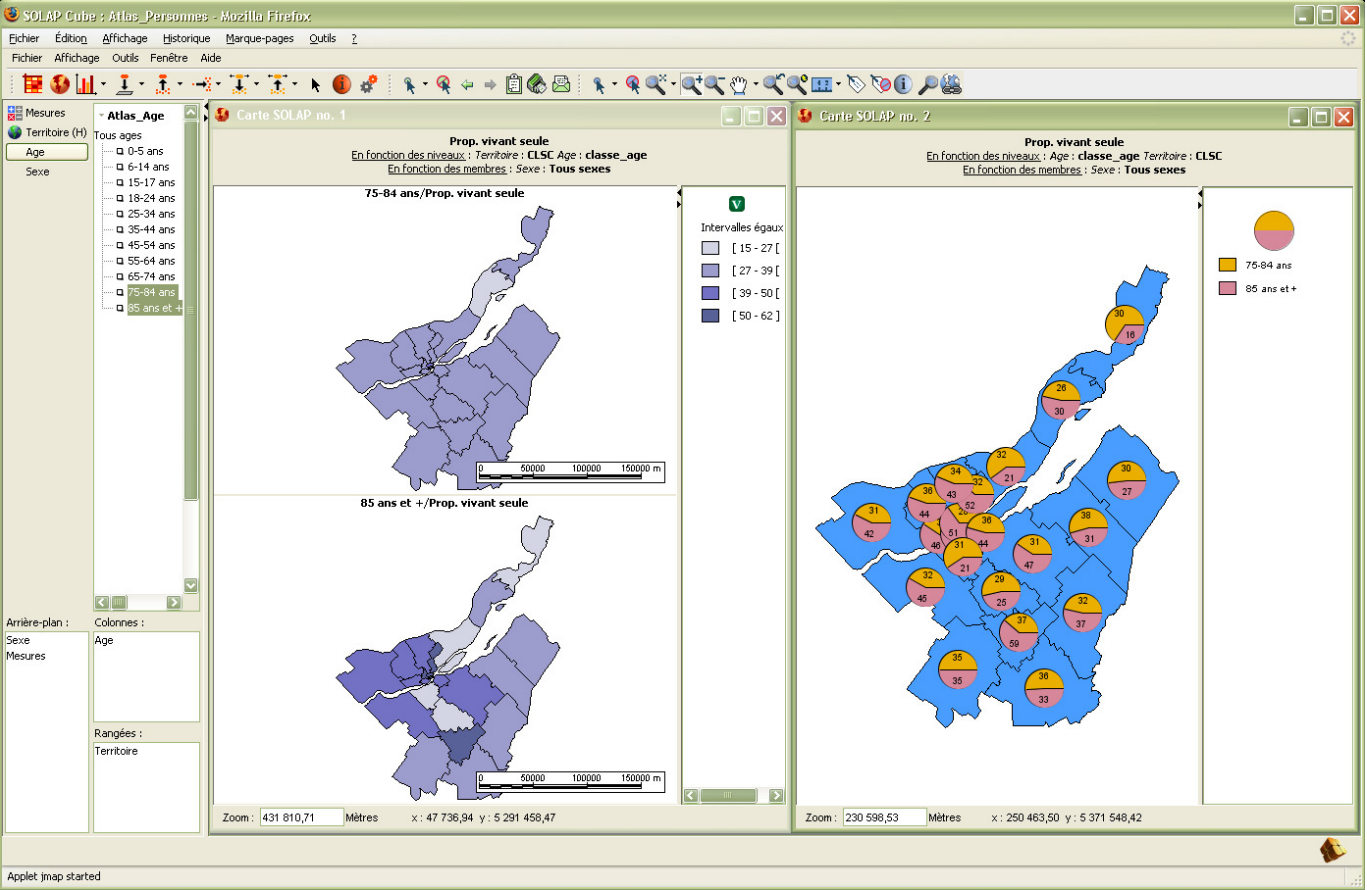
**Two different ways of spatially representing the proportion of the population living alone**. The left window displays the proportion for each age group at a specific LoD in a different map, while the right window uses pie charts over the territory to depict the ratio of the two age groups.

**Figure 5:**
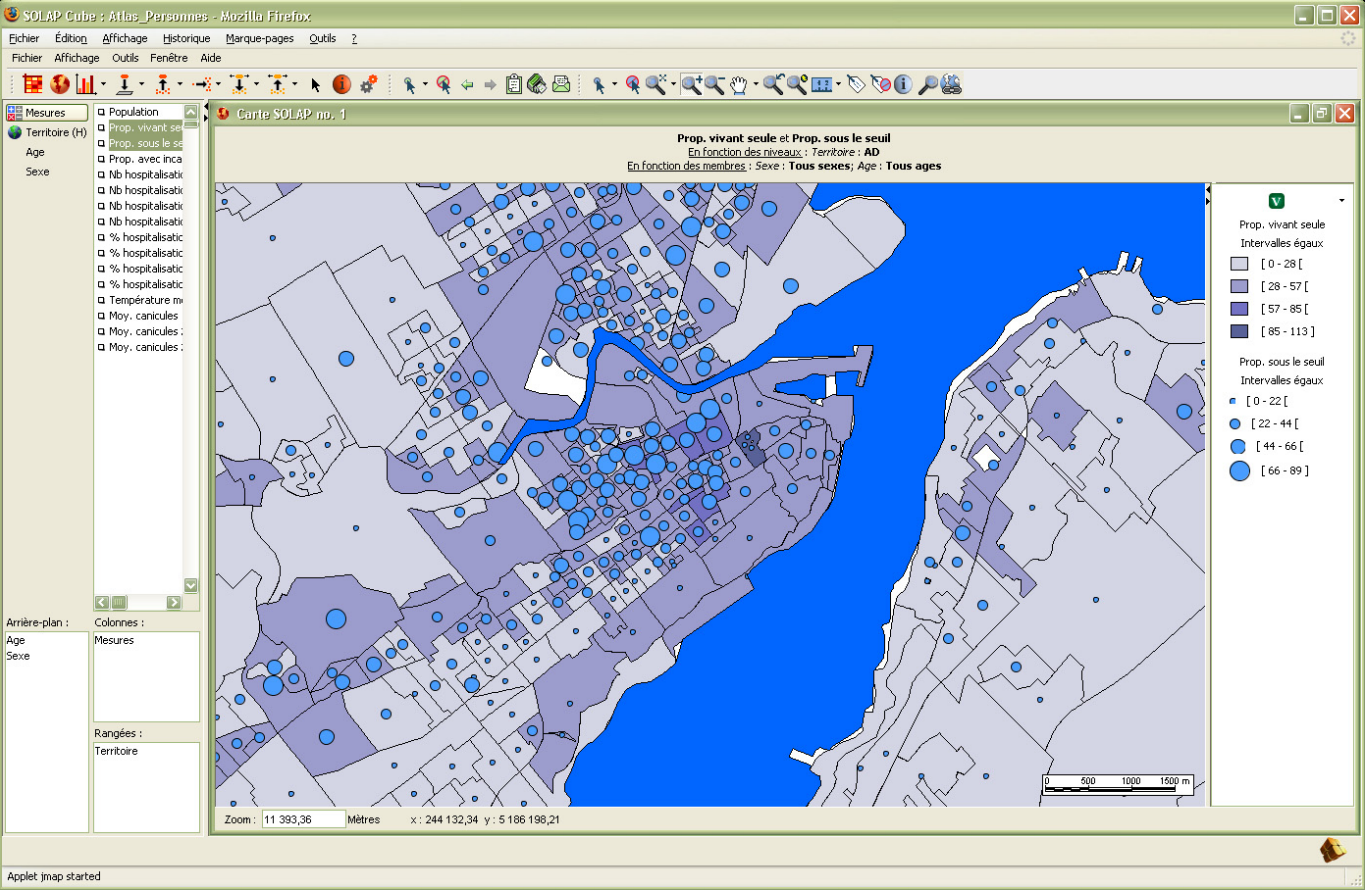
**Combining two measures on the same map for an enriched analysis**. Spatial representation of the proportion of the population living alone (choropleth map), combined with the proportion of the population with low income (symbols).

The first example deals with a part of the population that is potentially more vulnerable to an intense heat wave, namely elderly people living alone. Beginning with a global view of the situation, the user may spatially represent the proportion of people living alone at different levels of detail (LoD) regardless of gender and age (maps in Figure 3). Then, the user can get more insight by distributing this proportion according to population age (table in Figure 3) and filter the older ones (e.g., 75–84 years; 85 years or older) by simply selecting these groups directly on the selection tree (left part of the interface) (Figure 4). This analysis can be immediately enriched by adding a second measure, such as the proportion of the population with low income (shown using symbols over the choropleth map in Figure 5). Unfortunately, the level of detail of the data sources that were used to populate the datacube for this project (see the Methods section for further explanation) does not allow us to know whether the same person lives alone AND with a low income. However, such an analysis may be possible if the original data sources are made available to users. Besides, identifying the spatial regions where these two proportions are high may still be relevant and provide an indicator of more vulnerable zones. While similar results can be obtained with a GIS, it would require much more work regarding the development of the underlying spatio-temporal data structure, or the development of complex aggregative and temporal *ad hoc *SQL queries or of a predefined series of queries made available in a limited drop-down menu (typically including dozens of named queries with long and confusing names). In addition, data processing time would have been from a few seconds to several minutes in many cases, and it would have become too complex for many users. On the other hand, with the developed SOLAP application, it is a matter of a few mouse clicks, and the results are always displayed in 2 or 3 seconds. This allows an interactive, instantaneous exploration of data, whatever the level of detail involved, the time intervals, the number of themes that are cross-tabbed, or the types of data representation (one or several maps, tables and charts). Such exploration is not limited to a list of predefined queries since all allowed combinations of data are immediately available. As already stated by Codd [15], it is still nowadays recognized in the Business Intelligence community that no transactional system provides such capability. Furthermore, such capability is available to a larger number of users, thanks to a simpler user interface and new data exploration operators. This example shows that while a GIS and a SOLAP can produce practically the same end-results for surveillance, the major difference lies in speed and facility for the user. This difference is such that it truly encourages further analysis of data and more attempts at geographic knowledge discovery, while significantly reducing the time needed to produce reports.

**Figure 6:**
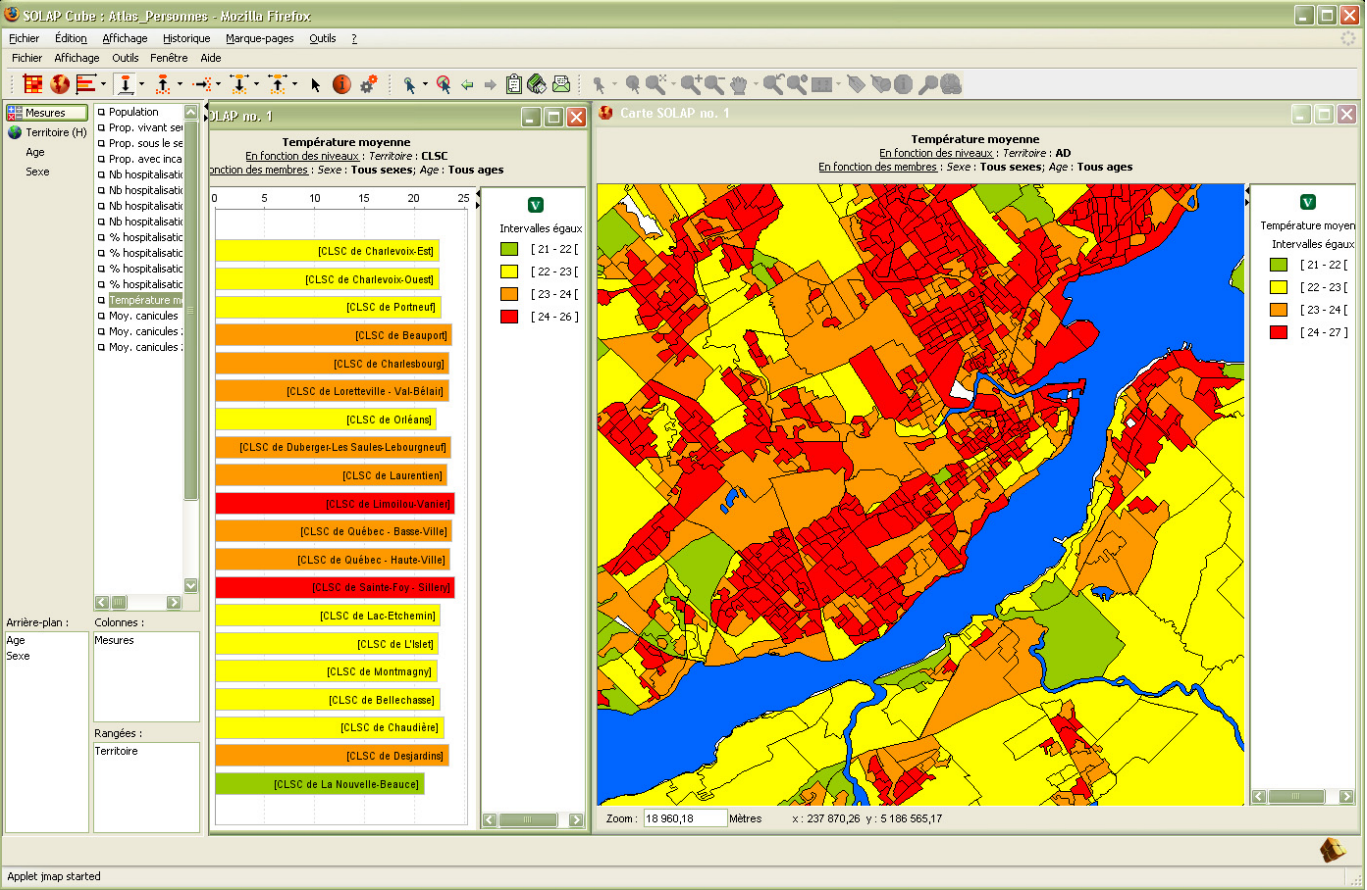
**Representation of the average temperature**. The table is used to compare the average temperature at a regional level (health regions), while the map represents this information at a more local level.

**Figure 7:**
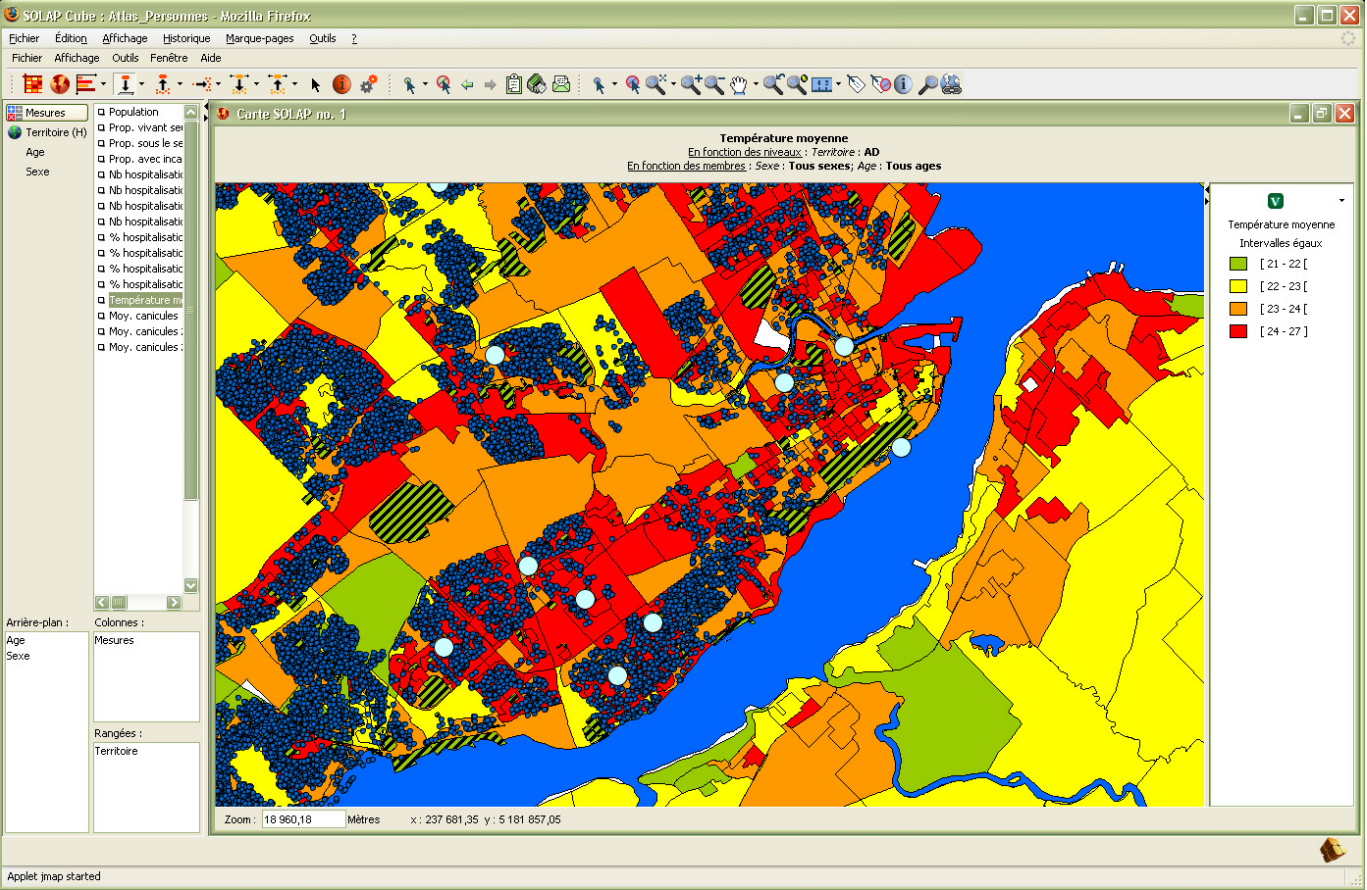
**Contextual spatial layers can provide additional information**. Québec City's public parks and private and public pools have been added to the temperature map at the local level.

Similar conclusions can be drawn from this second example of analysis which may be useful for improving a city's infrastructures in order to better respond to heat waves in a preventive context. First, the user can rapidly visualize the average temperature (obtained from Landsat satellite images taken in July 2007) over the territory at different levels of detail in order to detect hotter regions (Figure 6). From this representation, contextual spatial layers can instantly be displayed to provide additional information, whatever the level of detail being analyzed. Hence in Figure 7, public parks in Québec City (striped green polygons) as well as private (small blue symbols) and public (large blue symbols) pools can be displayed over the temperature map. Such a representation can help municipalities plan new green spaces or install new public pools to better serve the population. Furthermore, additional information can be easily combined, such as the ratio of hospitalization for respiratory diseases or the ratio of the population living with incapacity.

**Figure 8:**
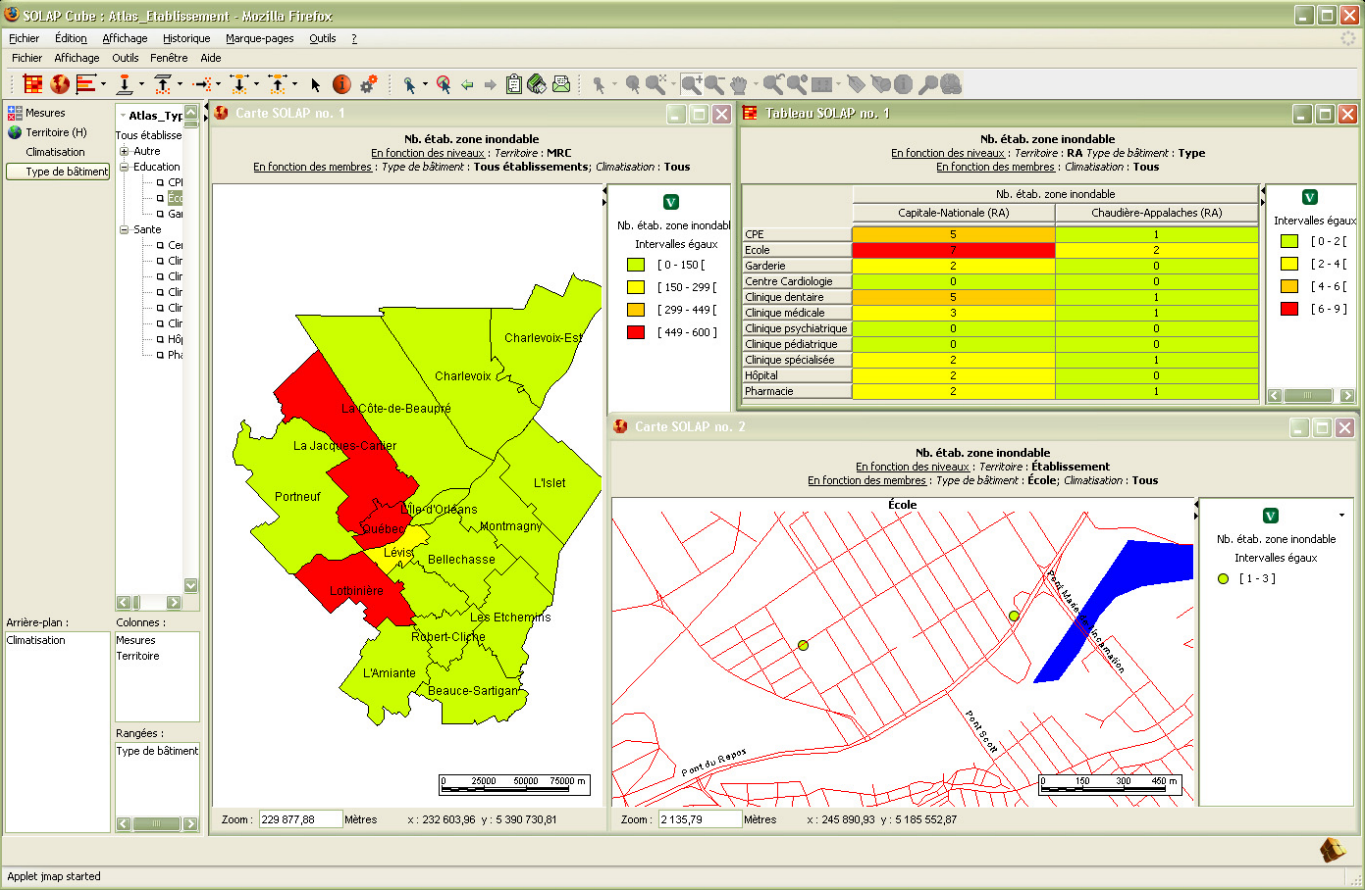
**The number of buildings located in areas with flooding potential**. Comparison of the number of buildings located in flooding areas at the county level (left map), at the regional level, and according to their type (table) and detailed spatial representation of the schools located in flooding areas (right map). The road network has been added as contextual spatial information.

A third example relates to the areas with flooding risks. The analysis may start with a cartographic representation of the number of buildings, whatever their category, located in flooding areas at the municipal level (left map in Figure 8), then a roll-up at the regional level while drilling down by building type (e.g., hospitals, schools, day-care centres) (table in Figure 8). Then, one can drill down to individually map only the schools located in a flooded area (right map in Figure 8) and add the road network to provide information useful in emergency situations (to plan and organize the rapid and efficient evacuation of vulnerable populations). All this takes about ten mouse clicks and 10 seconds of processing time. Several more surveillance examples could be presented from the developed system, including more complex data exploration and analysis, hypothesis building, hypothesis denial, emergency reaction planning, etc. Once again, while a GIS application could be developed and used to produce similar results, its efficiency from an end-user point of view would be far from the efficiency of the developed application.

### SOLAP, a new technology that raises new issues

In spite of offering a level of speed and ease of use not achievable by today's GIS, SOLAP technology also introduces new issues. First, it doesn't replace a GIS or spatially-enabled DBMS since SOLAP is not meant to support transactional (OLTP) activities such as operational data storing and integrity checking. In other words, for an organisation that collects and process its own data, SOLAP is considered as an add-on product rather than a replacement (thus involving additional cost and work) although it is not the case when an organisation uses only data from other agencies. Second, one must accept the fact that is well recognized by the Data Warehousing and Business Intelligence community that data will be duplicated. In other words, some data will be contained in several datacubes in addition to being contained in the data warehouse (if one's system architecture uses a data warehouse as explained in [23]). Indirect duplication also takes place when the result of aggregation calculations are stored, as it is the case with GIS and DBMS when views are materialized or result sets are stored in place of SQL query commands. It is the traditional optimization trade-off between speed vs storage where speed is favoured. This duplication of data leads to concerns about refreshing the SOLAP datacubes when the source databases are updated (remember that datacubes are read-only databases and can be fed solely from transactional database sources). Today's solution relies mostly on recalculating the datacubes periodically (e.g. every month, every survey) or when a given threshold is achieved (e.g. 2000 source updates). This additional process is necessary to allow adding the new data when there are enough updates to become meaningful for the finest grained level of aggregation in the datacubes while keeping past data for trends analysis. Nevertheless, as mentioned earlier, a more frequent and incremental addition of the new source data into the datacubes with incremental aggregation is possible. A third issue relates to the need for a development team to learn a different database paradigm, i.e. the paradigm related to multidimensional databases or datacubes. This has proven to be one of the most challenging issues insofar since the vocabulary, the concepts and the technology used in the Data Warehousing and BI community are typically unknown by the traditional OLTP database management community. One must remember that the same issue plagued the object-oriented world in the 1980's in spite of its numerous advantages. Finally, a last issue concerns the recent introduction of SOLAP technology in the commercial market. In spite of numerous commercial technologies appearing in the market over the last few months, including open-source offerings, it has not yet achieved the level of maturity that exist for non-spatial OLAP products with regard to ETL and OLAP server. A lot of research still goes on in university labs while the private industry works hard on delivering new spatially-enabled products targeted for 2009 and 2010.

## Methods used in the project

### The indicator phase

In the first phase of the project, we developed a series of surveillance indicators (Appendix 1 contains examples) in collaboration with end-users from health organizations (national health institute and regional health agencies) and municipal organizations (civil protection and land planning sectors). By applying a progressive convergence method, we were able to increasingly satisfy the needs expressed by the users and to adjust to foreseeable needs. We organized several meetings with these end-users based on a standardized semi-structured interview guide applicable in each of the meetings [24,25] in order to get them involved in the project and to validate the issues related to climate change. We thus obtained the formal support of the cities and health and social services agencies in the studied region (Québec City and Lévis, also called Québec City Metropolitan area). Since the project was meant to test the potential of SOLAP technology for surveillance needs and not to develop a full-blown system, we limited the field of the study to two subjects of interest (heat and water) and minimized the implementation risks related to the overall quantity of indicators and source databases.

To assess and validate the users' information requirements, we then prepared a list of questions relating to vulnerabilities to climate change for the two subjects retained, thus allowing the participants to express their comments on each of the proposed questions of interest, and to suggest other subjects, if necessary [26]. The themes addressed mainly dealt with the usefulness, for users, of accessing data related to known vulnerability risk factors (income, diseases, handicaps, lack of social network, geographical location, presence of specific services in the district, etc.) for different levels of spatial resolution (e.g., street, borough, city, region), as well as their interest in long-term regional climate simulations and demographic and health projections as a planning tool. We also documented the users' motivations regarding their answers for these putative indicators. All the users validated their needs by providing us with comments and suggestions; this included the rejection or modification of several of our proposals. Analysis of this information allowed us to develop an initial consensus list of priority indicators to be retained for the development of the first version of the system. These indicators are useful in both short-term emergency preparedness and risk prevention measures. The selection was made over a 2-month period and led to the deletion of about 50% of the indicators initially proposed by the research team, and the addition of a few proposed directly by users. The main reasons justifying these choices were the irrelevancy for their mandates and time (ex., being too far in the future, such as projections for 2080).

### The data integration phase

Once these indicators were defined, the second phase of the project was to collect data from sources among different organizations (provincial and federal departments, Health/Climate/Statistics/Natural Resources agencies) and to integrate them into a spatial multidimensional structure (also called spatial datacube), as typically done for analytical tools [27]. Several operations must be applied to the data sources to integrate them in a coherent manner into the same structure. These operations are typical of data warehousing architectures and are known as ETL (Extract, Transform and Load) processes [14,16,27]. When dealing with spatial data (such as with spatial data warehouse architectures), these processes become more complex and time-consuming since the spatial nature of the data brings several new issues that must be taken into account. For example, the data may be incompatible regarding different aspects such as their geodetic reference systems, measurement units, cartographic shape definitions, spatial resolution, symbolization, spatial accuracy, data format, temporal period and geometric evolution, to name a few. Since no traditional commercial ETL software can tackle these issues, this leads to a need for specialized integration and access tools to support the ETL phase, such as FME (Feature Manipulation Engine) [28], which provides operators for spatial data transformation and file translation. Though such tools can significantly facilitate ETL by allowing batch processes, there is always a need for "programming" such a tool as well as a need for manual operations that can only be done using GIS (Geographic Information System) tools. However, this ETL work is performed once to provide users with the power necessary for Spatial OLAP.

In this specific project, ETL processes were carried out to tackle the many incompatibilities between the spatial data sources. For example, we performed typical coordinate system translations and we geocoded data provided in an Excel file with Long/Lat coordinates or postal codes in order to produce point locations. We dealt with temporal incompatibilities regarding spatial boundaries that changed over the years, and differences in the definition of temporal periods for some indicators that vary depending on the spatial level of detail (LoD). For example, some indicators are defined for 2001 at the most detailed LoD of the spatial hierarchy, while they are defined for several years (from 1986 to 2050) at the more general LoDs. The present version of our application only considers the definition for 2001, but future versions will take semantic evolution into consideration. Fortunately, we did not have to aggregate detailed spatial data to produce the more general levels of the spatial hierarchy, since the cartography for each spatial layer already existed. Consequently, it only required that links between members of the different LoD be defined in order to support spatial drill-down/up operations. This task was relatively simple for upper LoDs because there was a perfect match between geometric boundaries since each level is conceptually defined in relation to the next one. However, this was not the case for the spatial members of the most detailed LoD. As a result, manual operations with ArcGIS (ESRI) were required to associate them with the higher LoD. This most detailed LoD has been included in the spatial dimension to provide the local analyses required for regional/municipal policy makers.

Following the ETL process, the data were integrated into a central repository according to the multidimensional (datacube) paradigm that governs most of today's decision-making applications [27]. Multidimensional structures, as opposed to relational structures typical of transactional systems (such as GIS), are denormalized (in relational database terms) in order to provide fast response times from a data structure that better matches the way users analyze their data (i.e., by interrelated thematic dimensions with multiple levels of granularity). This denormalization refers to the fact that summarized or aggregated data (which are data usually derived from existing ones) are typically calculated *a priori *and stored in the datacube structure so that they do not have to be recalculated each time they need to be accessed. This accelerates response times but creates database redundancy. Of course, designers have to find the right balance between what is stored and what can be calculated on the fly. For example, aggregating descriptive data (e.g., to calculate the number of people for a region from the sum of the sub-regions' population) is very easy and fast, whereas aggregating the spatial polygons of the sub-regions to form the boundary of the global region is more time-consuming and should be done *a priori *and stored in the database for performance reasons. Multidimensional structures are described in numerous books and papers and rely on concepts such as "*Dimensions*" (i.e., analysis themes like Age, Sex, Territory, Time), "*Measures*" (i.e., numeric values to be analyzed against dimensions such as Number of persons and Number of buildings), "*Facts*" (i.e., various combinations of dimensions and measures, like "There are 150 (measure) women (a member of the dimension Sex) older than 85 years of age (a member of the dimension Age), who live in Québec City (a member of the level City of the dimension Territory) in 2008 (a member of the level Year of the dimension Time)") and "*Cube*" (i.e., group of cross-tabbed and aggregated measures based on a group of dimensions). Interested readers can easily consult a detailed description of SOLAP concepts [29,7,6,11-13,16].

Decision-makers must have access to the most up-to-date information in order to make informed decisions. In several cases in public health, source data are gathered during specific periods (e.g., every year, every month) and pose no specific challenge to SOLAP applications other than refreshing the datacube (which takes from a few minutes to a few hours). Such an operation is typical of business intelligence applications. However, in other cases, source data are continuously updated; then, automatically propagating these updates in a multidimensional architecture as they happen is still difficult. Even today, it remains a research area due in part to their denormalized structures, which speed up analyses but increase data redundancy. Datacubes usually need to be entirely rebuilt each time new data comes in. On the other hand, when designing a datacube, strategic thresholds can be defined to reduce the impact of each source update on the aggregated datacube data and to initiate datacube recalculation only when updates have significant impacts on aggregated data (e.g., refresh after 10 updates, refresh every 6 hours only, refresh only after the total increases by 1% or more). This problem worsens with complex data, especially geospatial data, because they require complex and time-consuming operations on the geometry of spatial features. Reconstruction time generally increases dramatically with the size (i.e., the number of facts) and the complexity (i.e., number of dimensions, types of hierarchies) of datacubes. Therefore, entirely rebuilding the datacubes could result in an impossible avenue if time is a critical issue, such as in an emergency situation, and the geometries used are complex. Ways to incrementally propagate the updates in the datacubes therefore have to be investigated.

Updating techniques have been developed in this project and are based on rules for incremental and consistent updating processes that will only propagate updates on relevant data, and consequently scale down processing times [30]. These techniques are based on the "slowly changing dimensions" described by Ralph Kimball [31]. They have been adapted to the specific case of geometric dimensions. These incremental techniques were tested on a non-spatial datacube that contains 1,000,000 facts. Up to 50,000 updates, the time for an incremental updating of the datacube is rather constant and remains between 5 and 10 seconds. Afterwards, the processing time increases, but the amount of updates becomes quite unrealistic.

The performance of the incremental techniques adapted to spatial data was also tested for a datacube that contains (only) 50,000 facts. Again, the processing time for the incremental updating of the datacube is rather constant and remains between 5 and 10 seconds. Only 40 to 45 seconds are necessary in order to entirely rebuild the datacube. This is due to the small size of the datacube. However, it emphasizes that the differences between the processing times involved in these two techniques are important and will grow with the size of the datacube. In order to allow the propagation of updates stemming from possibly distributed OLTP sources into the data warehouse, these updating techniques have been implemented and deployed through a standardized Internet service. This service relies on the procedures presented by GeoKettle [32]. GeoKettle is an open source geospatial ETL tool developed by the GeoSOA research group of the Centre for Research in Geomatics (CRG) at Laval University. Specific transformations in the ETL tool have thus been designed in order to incrementally propagate updates in the datacubes, and are revealed through dedicated methods in the service contract: *listjobs() *lists all available transformations and provides details about each transformation, and *executejob() *starts a specific updating transformation. Updating procedures could thus be triggered remotely and in a standard way (based on the SOAP protocol) not only by a human but also by a machine. It then allows the machine-to-machine propagation of updates and a high level of automation in complex distributed architectures.

Updating techniques for geospatial datacubes still require research and development for their successful implementation in the context of surveillance. Nevertheless, results achieved in the project on the propagation of updates in geospatial datacubes are encouraging. They open doors for research on near real-time Spatial OLAP applications and on the integration of real time data flows stemming from distributed geo-sensors.

## Discussion

The application's strength in comparison to GIS applications for surveillance relies on the facility and rapidity with which decision-makers can analyze the different health vulnerability indicators that have been defined in this project. Through simple mouse clicks, these indicators can be studied at different levels of detail, for different epochs, compared, cross-tabbed, etc. This significant increase in interactiveness as well as immediate access to summarized data allows for more hypothesis building, faster response to emergency or public concerns, easier planning, shorter report building, rapid rumour denial, better understanding of global/local contexts, increased involvement of users, and so on.

The principal limitation of the developed datacubes relates to their structure, i.e., to the fact that several surveillance indicators are represented using measures instead of dimensions. This reduces flexibility during the analysis, since some information cannot be combined. For example, one cannot know at the present time whether the same person has respiratory disease and lives alone in a building that requires major repairs. This can be modified in future datacubes when designing final projects within the organizations involved (as opposed to a research project) because for this project, it was driven by a lack of access to private information (cf., confidentiality rules applying to the context of this project and authorizations considered too long to obtain within this 18-month project), by a limited budget (cf., detailed census or survey data, which are available only at high cost for such use), or in some instances, by the absence of such survey data to begin with. Obviously, this limit is related to the data sources and could be overcome by using finer granularity data already available in non-academic organizations. Nonetheless, the application provides access to numerous relevant indicators from a single gateway and illustrates the potential of the new technology for surveillance.

Regarding the method used for this project, we believe it was appropriate for such technology exploration. On the one hand, there was a hypothesis that surveillance needs for efficient multiscale-multiepoch-multitheme analysis matched well the capabilities of this new technology called SOLAP. On the other hand, there was a hypothesis that we could develop meaningful indicators making use of spatial referencing to help the targeted group of users. This led us to make a test with real data, to go through the whole process from analysis to design to implementation, and to gather comments of the involved users. In spite of a small number of developed datacubes and a lack of long-term day-to-day usage, we believe that the proposed approach and technology, although not statistically generalized, demonstrates the true potential of SOLAP technology to help specialists in climate-related health vulnerabilities surveillance (one must remember that it was not a full-scale implementation project but a GEOIDE or geomatics-funded research project). We have not analyzed yet the contextual factors that may influence our results during implementation (e.g. budget, time, resistance to change, available expertise), but we have delivered a solution received very positively from the involved partners and that no commercial technology insofar could achieve. This solution allows a health specialist to better understand the interactions between public health and climate change since it can be done interactively, providing very fast answers independently of the complexity of the query and after only a few mouse clicks. This also facilitates decision-making since one doesn't have to wait for a GIS specialist to be available and to start a time-consuming process every time more global information is looked for. Consequently, we believe that our goal has been achieved: to validate the potential of such new technology for public health surveillance. At the same time, we are aware that our conclusion would be more convincing if (1) a larger number of projects could be tested and the results compared query-by-query with existing GIS and spatially-enabled DBMS on a Return-On-Investment basis (2) a detailed analysis of the types and quantity of queries performed over a long period of time would support the need for rapidly and easily available summarized information (although our analysis of existing reports convinced us of such a need), and (3) the large number of OLAP users presently working in public health could complain about the lack of a spatial component to improve their analysis or the large number of health-related GIS users could complain about the lack of efficient summarizing, comparative and interactive analysis capabilities.

**Figure 9:**
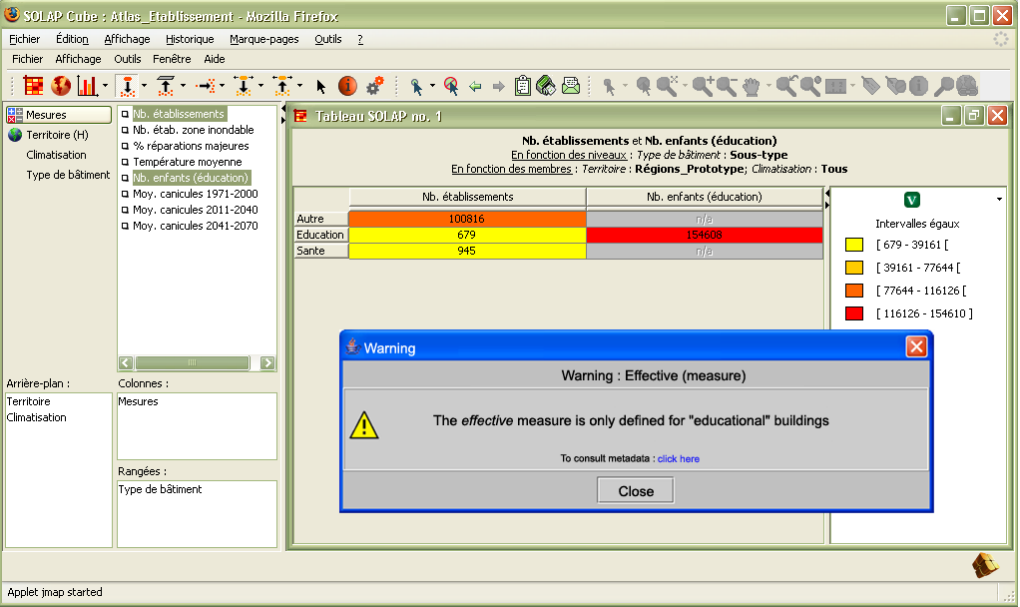
**Contextual warnings will be used to provide additional information and avoid data misinterpretation**. Contextual warnings can be displayed during the analysis to inform the user about implicit aspects of the data that must be taken into account when conducting the analysis to avoid data misinterpretation.

From this project perspective, the next steps will include the integration of prediction data about future demographic evolution and population health. In addition, we will also continue working on reducing the risk of potential misuse of some combinations of databases through warnings for users (figure 9). This is a consequence of allowing increased analytical power and access to a larger group of users. Although data exploration constraints were implemented in the initial datacube design, the fact that some meaningless combinations of data would be left was almost inevitable (and was in fact a research item in this project, see [33,34]). Now that the method for managing the risks related to potential misuse has been developed, these results will be considered for future developments. Finally, more development should take place regarding the real-time transmission of source data updates and their impacts on datacube refreshing when needed.

## Conclusion

Transactional systems such as GIS and DBMS are widespread in surveillance activities and play a fundamental role in storing, organizing, validating, protecting and disseminating data. They also play an important role in efficiently answering simple queries. However, they fall short when users require *ad hoc *multi-scale, multi-epoch, multi-theme complex queries with immediate answers, or when they require interactive exploration of their data for geographic knowledge discovery or hypothesis building and validation. On the other hand, Business Intelligence technologies have been designed to support such complex analysis and knowledge discovery and have been extended to spatial data in the last few years, leading in particular to the development of Spatial OLAP as a technology to be used complementarily with GIS. Through a concrete application, this paper has demonstrated the potential of Spatial OLAP to support the interactive exploration and analysis of geo-referenced indicators relevant for the monitoring of climate change impacts on population health and well-being. Using simple navigation operators, specialists and decision-makers are able to create, with much less effort and time, a multitude of views (tabular, graphic or cartographic) to support their analyses, and to navigate through levels of information detail in more efficient ways.

Providing such capabilities using dedicated technology rather than tweaking traditional technologies not meant for such purposes allows an organization to deliver better results in shorter periods and within smaller budgets. Non-spatial Business Intelligence technology from Oracle, Microsoft, Cognos, Business Intelligence and others is already widespread, including in public health; this is also true for GISs. Bridging the gap between these two technologies, as tested in this project simply facilitates complex spatio-temporal analysis and geographic knowledge discovery. It is the only way to accelerate multi-scale spatio-temporal analysis to a point where a user can continuously keep her/his train of thought by focussing on "what" is wanted rather than "how" to get it. In particular, SOLAP uses restructured subsets of GIS data to perform *ad hoc *analyses, which are tedious and complex to perform with GIS. It can be seen as a powerful GIS extension that provides new capabilities in terms of power and ease of use, as well as operators not supported by GISs. SOLAP is improving (not replacing) GIS in the same way that OLAP improved (not replaced) DBMS. The newly provided capabilities were unanimously recognized by the external participants in this project as highly desirable for decision-makers to efficiently take into account existing public health vulnerabilities to climate change when budgeting, designing and localizing interventions aimed at adaptation and the prevention of risks.

## Competing interests

The authors declare that they have no competing interests.

## Authors' contributions

All authors were involved in the conception and design of the application as well as in the drafting and revision of the manuscript. EB was also responsible for data integration and programming.

## Appendix 1

Some indicators to help define vulnerabilities to climate change were retained by the participants and included in the prototype. These indicators address the following questions for either medium- or long-term health protection purposes, under the Heat and Water themes. The questions of interest for those participants were:

### A: MEDIUM-TERM HEALTH PROTECTION; THEME HEAT

- How many people older than 65 years and younger than 5 years live in a specific geographic area? What is the likely demographic trend over the next 25 years?

- How many people with cardiovascular, respiratory, neurologic and psychological diseases live in a specific geographic area?

- How many people with incapacity live in a specific geographic area?

- How many people live alone in a specific geographic area, by age group?

- How many people with low income live in a specific geographic area?

- How many buildings require major repairs in a specific geographic area?

- Where are the day-care centres and how many children do they care for, in a specific geographic area?

- Where are the schools in a specific geographic area? Do they have air-conditioning?

- Where are the health-care centres in a specific geographic area?

- Where are the areas that more sensitive to intense heat waves in a specific geographic area due to urban heat islands?

- How many people live in urban heat islands in a specific geographic area?

- How many people don't have access to a public pool within a 1-km radius?

- How many people don't have access to a beach accessible by public transit?

- How many people don't have access to a park or green areas within 500 m of their home?

### B: MEDIUM-TERM HEALTH PROTECTION; THEME WATER

- Where are the urban spots that are more sensitive to heat waves, intense rain, flooding or droughts in a specific geographic area?

- How many public buildings are located in flooding areas?

- Which health-care buildings are located in flooding areas?

- Which day-care centres are located in flooding areas?

- Which schools are located in flooding areas?

### C: LONG-TERM HEALTH PROTECTION; THEMES HEAT AND WATER

- How many heat waves will there be in 2025 and 2050 according to Regional Climate Models?

- How many people will there be in 2025 and 2050 in a specific geographic area (demographic trends)?

- How many people with cardiovascular, respiratory, neurologic and psychological diseases will there be in 2025 and 2050 in a specific geographic area according to current demographic and sanitary trends?
